# Evaluation of Buruli Ulcer Disease Surveillance System in the Ga West Municipality, Ghana, 2011–2015

**DOI:** 10.1155/2019/4721236

**Published:** 2019-11-12

**Authors:** Tanko Rufai, Enoch Aninagyei, Samuel Oko Sackey, Ernest Kenu, Edwin Andrew Afari

**Affiliations:** ^1^Field Epidemiology and Laboratory Training Programme, Department of Epidemiology and Disease Control, School of Public Health, College of Health Sciences, University of Ghana, Accra, Ghana; ^2^Ghana Health Service, Accra, Ghana

## Abstract

**Background:**

Buruli ulcer (BU) is one of the most neglected tropical diseases caused by *Mycobacterium ulcerans*. *M. ulcerans* infection may manifest initially as a pre-ulcerative nodule, a plaque, or oedema which breaks down to form characteristic ulcers with undermined edges. The Ga West Municipality is an endemic area for Buruli ulcer, and we evaluated the BU surveillance system to determine whether the system is meeting its objectives and to assess its attributes.

**Materials and Methods:**

We used a checklist based on Centers for Disease Control and Prevention (CDC) updated surveillance evaluation guidelines, 2006. We reviewed records and dataset on Buruli ulcer for the period 2011–2015. The evaluation was carried out at the national, regional, district, and community levels using the Ga West Municipality of the Greater Accra Region as a study site. Interviews with key stakeholders at the various levels were done using an interview guide, and observations were done with a checklist. Data were entered and analyzed using Epi info 7.

**Results:**

A total of 594 cases of Buruli ulcer were reported from 2011 to 2015 in Ga West. The number of confirmed cases decreased from 109 in 2011 to 17 in 2015. The system was useful, fairly simple, flexible, representative, and fairly acceptable. The system was sensitive with a PVP of 45.3%. Although the data quality was good with 85% of case report forms completed, there was under-reporting (3.6%), some discrepancies of data at the district, regional, and national levels. The system was moderately stable, and timeliness of reporting was 30.7%.

**Conclusion:**

The Buruli ulcer surveillance system is meeting its set objectives, and the data generated are used to reliably describe the epidemiologic situation and evaluate the results for actions and plan future interventions. There is a need for timely submission of data. We recommend that the National Buruli Ulcer Control Program (NBUCP) provides logistical support to treatment centres.

## 1. Introduction

Buruli ulcer (BU) is a neglected tropical disease caused by *Mycobacterium ulcerans* and is characterized by a chronic necrotizing, ulcerative lesions of the skin [1]. *M. ulcerans* infection may manifest initially as a pre-ulcerative nodule, a plaque, or oedema which breaks down to form characteristic ulcers with undermined edges [2]. It is the third most widespread Mycobacterium infection after tuberculosis and leprosy causing morbidity in immunocompetent humans worldwide [3]. The virulence of *M. ulcerans* is dependent on mycolactone, a lipid toxin with cytotoxic or immunosuppressive properties depending on its concentration. The exact mode of transmission of *M. ulcerans* is still unknown [4]. Studies have shown that BU is commonly found in populations living near rivers, swamps, and wetlands [4–6]. In several instances, local environmental events, such as deforestation, flooding and building of dams, or agricultural activities such as irrigation, have been associated with the emergence of BU [5, 6]. At least 33 countries with tropical, subtropical, and temperate climates have reported Buruli ulcer in Africa, South America, and Western Pacific regions [3]. In 2015, 2037 new cases were reported from 13 countries to WHO. In 1999, a national survey conducted in Ghana on the prevalence of BU recorded about 6000 cases [1]. The World Health Assembly adopted a resolution in 2004, which called for increased surveillance, control, and intensified research to develop tools for diagnosis, treatment, and prevention of BU. From 2000-2001, The National Buruli Ulcer Control Programme (NBUCP) was established by the Ministry of Health, Ghana, with an objective to minimize the morbidity and disability associated with Buruli ulcer disease, collaborate with research centres in diagnosis and case management, and standardized case management with antibiotics, surgery, and prevention of disability. Though Buruli ulcer disease is not usually fatal, it leads to profound morbidity especially in areas where treatment options are limited. The large ulcers often lead to scarring, contractual deformities, amputations, and irreversible disabilities; thus, a surveillance system was set up to monitor the impact of Buruli ulcer interventions in terms of incidence and prevalence of the disease. Regular and relevant evaluation of this system is critical in order to improve their performance and efficiency; hence, we evaluated the surveillance system to see whether it is meeting its objectives and to assess its attributes and usefulness.

## 2. Methods

### 2.1. Study Setting

**Figure 1:**
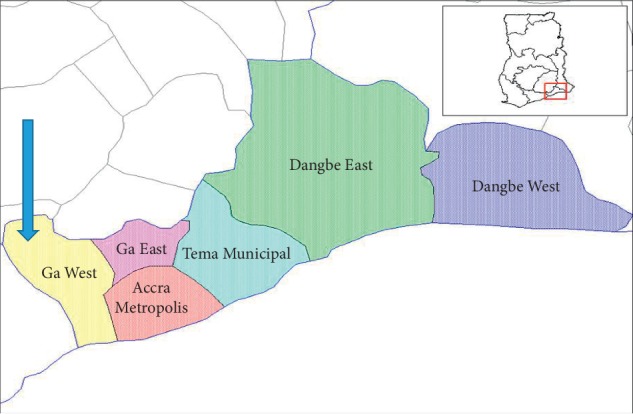
Map of Greater Accra showing Ga West (greater Accra districts maps of net) [8].

Ga West Municipality is one of the sixteen districts in the Greater Accra Region, carved out of the erstwhile Ga district which was created in 1988. The district is 60% rural and 40% periurban and urban. It is made up of about 150 communities with Amasaman as its district capital. The Ga West Municipality shares boundaries with the Ga East and the Accra Metropolitan Area to the East, Akwapim South to the North, Ga South to the South, and Ga Central to the North-South. It occupies a total land surface area of 299.578 square kilometres. The population of the municipality as of 2015 was 262,742 [7]. Currently, the municipality is divided into three submunicipal areas for the purpose of planning and delivery of services, namely, Amasaman, Ofankor, and Pokuase. The municipality has a district hospital, four health centres, three clinics, four community-based health planning services (CHPS) compounds, 9 urban CHPS, 10 private hospital/clinic, and 4 maternity homes. Ga West is an endemic municipality in Buruli ulcer cases, and as a result, there are eight BU treatment centres with the municipal hospital serving as the referral point not only for the municipality but also the entire Greater Accra Region and neighbouring regions for severe cases of BU management. There is a ward in the municipal hospital in charge of BU treatment such as antibiotic treatment, surgery, wound debridement, and dressing (Figure 1).

### 2.2. Study Design

The evaluation was carried out at the national level using the Buruli Ulcer Control Programme, the regional level using the Greater Accra Region, the district, and community levels using the Ga West Municipality in January 2016–March 2016. A semistructured interview guide, checklist based on the Centers for Disease Control (CDC), updated guidelines for Evaluating Public Health Surveillance Systems, 2006 [9], and the direct observation method was used to collect data at the national, regional, and district levels. Stakeholders interviewed included the National Buruli Ulcer Control Programme Director, Regional Director of Health Services, and the Deputy Director of Public Health in GAR, Regional Surveillance Officer, District Health Director, District BU Coordinator, and some Community-Based Surveillance Volunteers (CBSVs) in the district. We reviewed data from the District BU register, BU02 registers from some treatment centres, and District Health Information Management System (DHIMS). We collected secondary data for the period 2011–2015. We then assessed the performance of the system and its attributes such as simplicity, flexibility, data quality, acceptability, sensitivity, predictive value positive, representativeness, timeliness, and stability. Data were cleaned and analyzed using Epi Info version 7™ to generate frequencies, proportions, and graphs.

## 3. Results and Discussion

### 3.1. Stakeholders

The stakeholders of NBUCP at the national level include the WHO, Central Government through the Ministry of Health, Ghana Health Service, Korle-Bu Teaching Hospital (KBTH), Clinicians and other medical personnel, all health and treatment centres, Research Institutions (NMIMR, KCCR), and Non-Governmental Organizations (NGOs) like the America Leprosy Mission (ALM). At the regional and district levels, the local government through the District Assemblies, Chiefs, and people of the community and some NGOs like World Vision International, Anesvad, from Spain. Most of the stakeholders provide logistical and financial support to the program for its activities. The research institutions support the national control programme with case confirmation. The BU surveillance system in Ga West Municipality has a standard case definition, and because ulcers and nodules are easily identified, it makes the case definition simple to detect cases.

### 3.2. Case Definition

#### 3.2.1. Suspected Case

A person presenting a painless skin nodule, plaque, or ulcer, living or having visited a BU-endemic area.

#### 3.2.2. Confirmed Case

A suspected case confirmed by at least one laboratory test (ZN for AFB, PCR, culture, or histology).

### 3.3. Operation of the System

**Figure 2:**
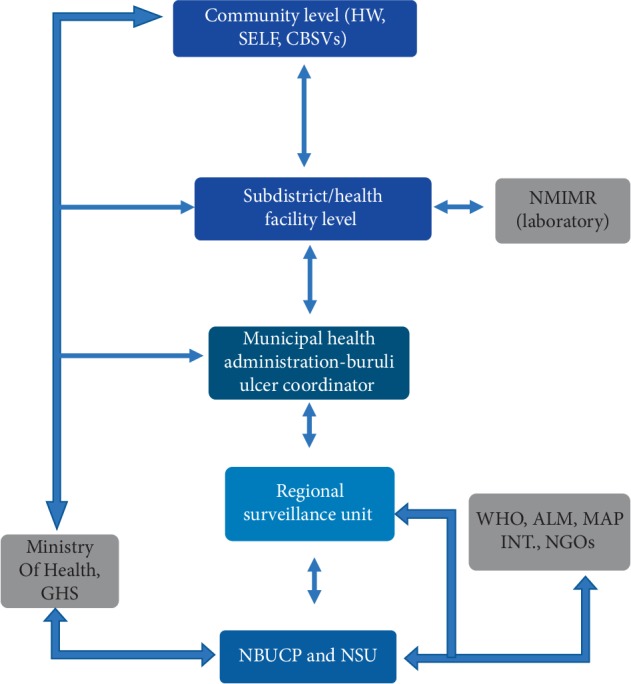
Flow chart of Buruli ulcer surveillance system.

Data on this system are collected mainly through a combination of passive reporting and active reporting. At the community level, health workers or CBSVs detect cases of BU and report to the health facility, active case searches during home or school visits. Some patients also report themselves. At the health facility level, diagnosis of Buruli ulcer depends on clinical presentation and laboratory confirmation. On Wednesdays, which happens to be “a clinic day” for Buruli ulcer, samples are taken from new patients by staff of Noguchi Memorial Institute for Medical Research for laboratory confirmation and registration of cases in the district BU register (BU-02 Form). Results of the test are ready after a week. Health facilities extract information from BU-01 case registration form or from the BU register to the monthly BU-02 form and deliver hard copies to the Municipal Health Directorate (MHD), precisely to the District BU Coordinator at the end of every month. No analysis of data is done at this level. The coordinator then compiles all the cases from the facilities on another BU-02 form which is reported quarterly to the regional surveillance unit and NBUCP through e-mail and hardcopy. Copies are stored in the computer and external drive. The District Disease control officer also compiles the Integrated Disease Surveillance and Response (IDSR) monthly reports and submits to the regional surveillance unit while soft copies of the monthly morbidity return forms are sent to the Health information unit every month. Soft copies are stored on computers and hard copies in files at the office. Data analysis is as well carried out at this level using Microsoft Excel to provide information to all the stakeholders in the district for action. At the regional level, the regional surveillance officer compiles all reports received from the districts. Data analysis is done to assess the trend and to give information to the regional Director of public health as well as the district Directors. The IDSR monthly report is then sent to the National Surveillance Unit (NSU) on a monthly basis while BU-02 quarterly forms are sent to NBUCP on quarterly basis. Feedback is sent to the districts in the form of emails, telephone calls, review meetings, and annual reports. At the national level, the surveillance unit of the NBUCP receives data from district and regions. Data analysis is conducted to generate age, sex, district, and regional distributions. Data analysis is carried out to generate the BU categories, clinical forms, suspected and confirmed BU cases, and trends of new and recurrent cases. After analysis of the data, feedback is sent to the regions and districts quarterly through e-mail and annual reports. At the end of each year, reports containing the total number of BU cases and the various indicators in Ghana are sent to WHO during the annual meeting in Geneva. The flow of information from one level to the other is shown in Figure 2.

### 3.4. Resources Used to Operate the System

At the national level, NBUCP has a staff strength of five and one employed by an NGO to assist the program. NBUCP has strong collaboration with the research centres for confirmation of cases. At the regional, district, and facility levels, the same officers are used for all public health and disease control activities. The integration with the health service surveillance system makes BU surveillance system less expensive to run. The main sources of funding for NBUCP include the Government of Ghana through Ministry of Health (MOH), WHO, America Leprosy Mission, NGOs such as Anesvad.

## 4. Performance of the BU Surveillance System

### 4.1. Usefulness

**Figure 3:**
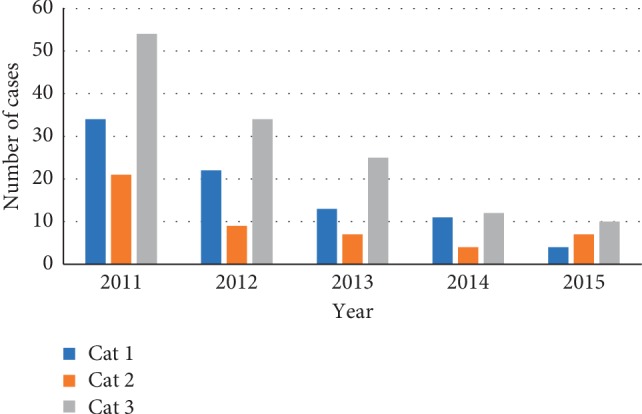
Categories of Buruli ulcer cases in Ga West, 2011–2015.

The Buruli ulcer surveillance system in Ga West is a vital source of information. The data are useful for understanding the severity of the disease and for planning and monitoring the impact of interventions put in place to minimize the morbidity and disability associated with the disease (Table 1). Due to the high number of cases recorded in 2011-2012, there was support from an NGO (Anesvad) which led to the introduction of six new treatment centres in 2013/2014. This brings the health facilities capable of providing dressing and antibiotic to BU patients to eight. From 2011–2015, a total of 269 confirmed cases of BU at various stages were identified. According to Johnson [10], Buruli ulcer is usually not fatal but leads to profound morbidity especially the category II and III ulcers which can lead to permanent disability. A relevant measure of early reporting is the size of the lesion, which is reflected by the WHO categorization system for BU. The lesions are group into three categories based on size by WHO: thus, category I, ≤5 cm in diameter, category II, 5–15 cm in diameter, and category III, >15 cm in diameter. Patients commonly present with large lesions, with 135 (50.6%) of category III, 84 (31.4%) category I, and 48 (18.0%) category II (Figure 3). There was 61.7% treatment success without limitation of movement to the affected part of the patient making the system very useful. This could be due to interventions such as increased in number of treatment centres, construction of physiotherapy department, special clinic day (Wednesday) for BU patients, and availability of Buruli ward. For the years under evaluation, there was a decrease in the number of cases. This could be due to an increase in awareness of the disease.

## 5. System Attributes

### 5.1. Simplicity

We assessed simplicity by the level of easiness for detection of cases and amount of follow-up that is necessary to update data on the case. Even though there is clarity in the case definition, the system was found not simple because confirmation of cases by the laboratory takes a week. Throughout the various levels of reporting, (8/10) of respondents complained of too much variable needed to fill the BU-02 form.

### 5.2. Flexibility

Flexibility was assessed by determining the surveillance system's ability to adapt to new demands such as the integration with other diseases on the IDSR. The system was found to be flexible because it was well integrated with other diseases like Leprosy, Leishmaniasis, and Yaws.

### 5.3. Data Quality

Data quality was assessed by examining the percentage of “unknown” or “blank” responses to the items on the BU02 forms and review of sampled data. According to the report by WHO [11], under-reporting exists within countries, and in the current study, 85% of case report forms were completed with 3.6% under-reporting. There were discrepancies of data at all levels: district, regional, and national.

### 5.4. Stability

The systems were seen to be fairly stable partly because it makes use of the Ministry of Health/Ghana Health Service staff to collect data and manage cases. It was found that it depends heavily on NGO's support and research institutions for confirmation of cases. Challenges with logistics, transport, and communication were also apparent during the evaluation.

### 5.5. Acceptability

All the public sector health facilities in the districts submit reports to the municipal health directorate. However, it was apparent that the private sector does not report and hence are not part of the surveillance network.

### 5.6. Representativeness

The surveillance system was representative in person, place, and time. The surveillance system collected data all year round from all the subdistricts. Over the five-year period, all persons were under surveillance. Cases were reported with variables: sexes, all ages, residence, clinical forms, location of lesion, and category of lesion. Cumulatively, 53.9% of cases were males, and children ≤15 years were 30.5% within the time period.

### 5.7. Sensitivity

**Figure 4:**
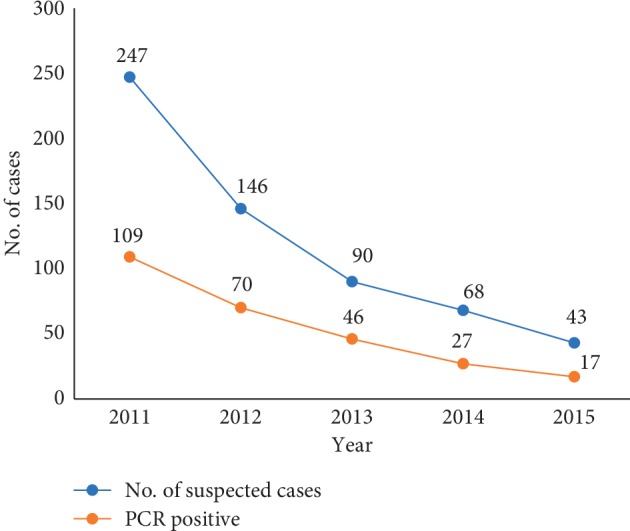
Trend of suspected and confirmed cases in Ga West, 2011–2015.

We assessed sensitivity by the ability of the system to pick cases. According to the clinical case definition, a total of 594 suspected cases were identified during the period and confirmed by Noguchi Memorial Institute for Medical Research (NMIMR) using polymerase chain reaction (PCR) detection of the insertion sequence IS*2404* (Figure 4). It shows the ability to monitor changes over time.

### 5.8. Predictive Value Positive (PVP)

From 2011–2015, of 594 suspected cases, 269 were confirmed by PCR as positive. This gave a PVP of (269/594) × 100 = 45.3%

### 5.9. Timeliness

Timeliness was calculated based on the BU records from the District Health Information Management System. The sub-facilities are supposed to report on the 5^th^ of the ensuing month, and the district BU coordinator/health information officer is supposed to enter the data into DHIMS on 15^th^ of every month. After this date, the DHIMS captures it as late entry. Even though there was an improvement on the timeliness of reporting, on the average, the system was not timely (Table 2).

## 6. Conclusion

In conclusion, the surveillance system in Ga West is meeting its set objectives and is useful. However, data quality, timeliness, and private participation are a challenge. It depends heavily on NGO's support and research institutions for confirmation of cases which could affect its stability.

We, therefore, recommended that, the National Buruli Ulcer Control Program Director should provide regular logistical support for all treatment centres and bring private health facilities into the surveillance network. The district BU coordinator should always crosscheck for discrepancies in the data generated, reconciled with the disease control officer in charge of integrated disease surveillance and response monthly forms.

## Figures and Tables

**Table 1:** Sex, age group, and clinical forms of BU seen in Ga West, 2011–2015.

Year	Sex		Age group		Clinical forms of cases seen				
	Female	Male	<15 yrs	15 yrs and above	Ulcer	Plaque	Oedema	Mixed forms	Nodule
2011	42	67	31	78	92	4	2	9	2
2012	32	38	19	51	63	1	1	4	1
2013	23	23	18	28	36	2	0	6	2
2014	16	11	8	19	23	1	1	0	2
2015	11	6	6	11	17	0	0	0	0
Total	124 (46.1%)	145 (53.9%)	82 (30.5%)	187 (69.5%)	231 (85.9%)	8 (2.9%)	4 (1.4%)	19 (7%)	7 (2.6%)

**Table 2:** Timeliness of reporting BU cases in (DHIMS).

Year	Timeliness (%)	Average (%)
2011	0	30.7
2012	0	
2013	14.3	
2014	39.3	
2015	100	

## Data Availability

The data used to support the findings of this study have been deposited in the Harvard Dataverse repository (https://doi.org/10.7910/DVN/HLN9L9).
